# Syringomyelia Charcot-arthropathy involving the joints of both hands

**DOI:** 10.1007/s10067-025-07436-6

**Published:** 2025-04-28

**Authors:** Jiaojiao Cao, Di Zhang, Jianguo Yang, Bing Fan

**Affiliations:** 1 The First School of Clinical Medicine, Shandong University of Traditional Chinese Medicine, Jinan, Shandong China; 2 Rheumatology and Immunology Department, Affiliated Hospital of Shandong University of Traditional Chinese Medicine, Jinan, Shandong China

A 72-year-old woman presented with a 30-year history of progressive left elbow and bilateral hand joints deformities, accompanied by scoliosis (Fig. 1). Multiple evaluations failed to establish definitive diagnosis or therapies, with symptom management limited to NSAIDs. Clinical examination revealed fixed flexion contractures of the left hand impairing fist formation, limited left elbow mobility, and thick fingers. Notably, the patient reported the absence of redness, pain, morning stiffness, or systemic symptoms (fever, rash, Raynaud’s phenomenon, oral ulcers) since disease onset. Serological tests showed negative RF, CCP, ESR, CRP, HLA-B27, ANA, and ANCA, along with normal IGF-1 levels, excluding inflammatory arthritis (RA, AS, PsA), connective tissue diseases, vasculitis, and acromegaly. Upon re-evaluating the medical history, the patient reported a progressive decrease in superficial sensation in the left upper limb. “Syringomyelia Charcot-arthropathy” was diagnosed after a cervical MRI showed Chiari malformation and syringomyelia (Fig. 2). Charcot arthropathy (CN), a neuropathic osteoarthropathy caused by sensory and autonomic dysfunction, classically manifests with painless joint swelling, progressive deformity, and bony crepitus [1]. In the late stages, X-ray imaging demonstrates joint disorganization, osseous fragmentation, subluxation, and mixed osteolysis and sclerosis. While typically involving proximal joints (shoulders, elbows), this case exhibited rare small-joint (interphalangeal) involvement, mimicking destructive inflammatory arthritis. This atypical presentation underscores the necessity of comprehensive differential diagnosis. Notably, radiographic joint damage severity discordant with clinical symptoms was observed. This paradox can be attributed to the underlying syringomyelia (SM), which drives CN pathogenesis through a dual neuropathic mechanism. SM, characterized by segmental dissociated sensory loss and autonomic dysfunction, originated from Chiari malformation-induced craniocervical CSF obstruction, forming a syrinx. The syrinx compressed spinal cord posterior horns, disrupting proprioceptive and nociceptive signaling, thereby impairing microtrauma perception. Combined with autonomic dysregulation-driven hyperemia and osteoclast activation, this triggered repetitive joint stress, chronic inflammation, and accelerated bone resorption—explaining severe structural damage progression despite minimal symptoms [2, 3, 4].

**Fig. 1:**
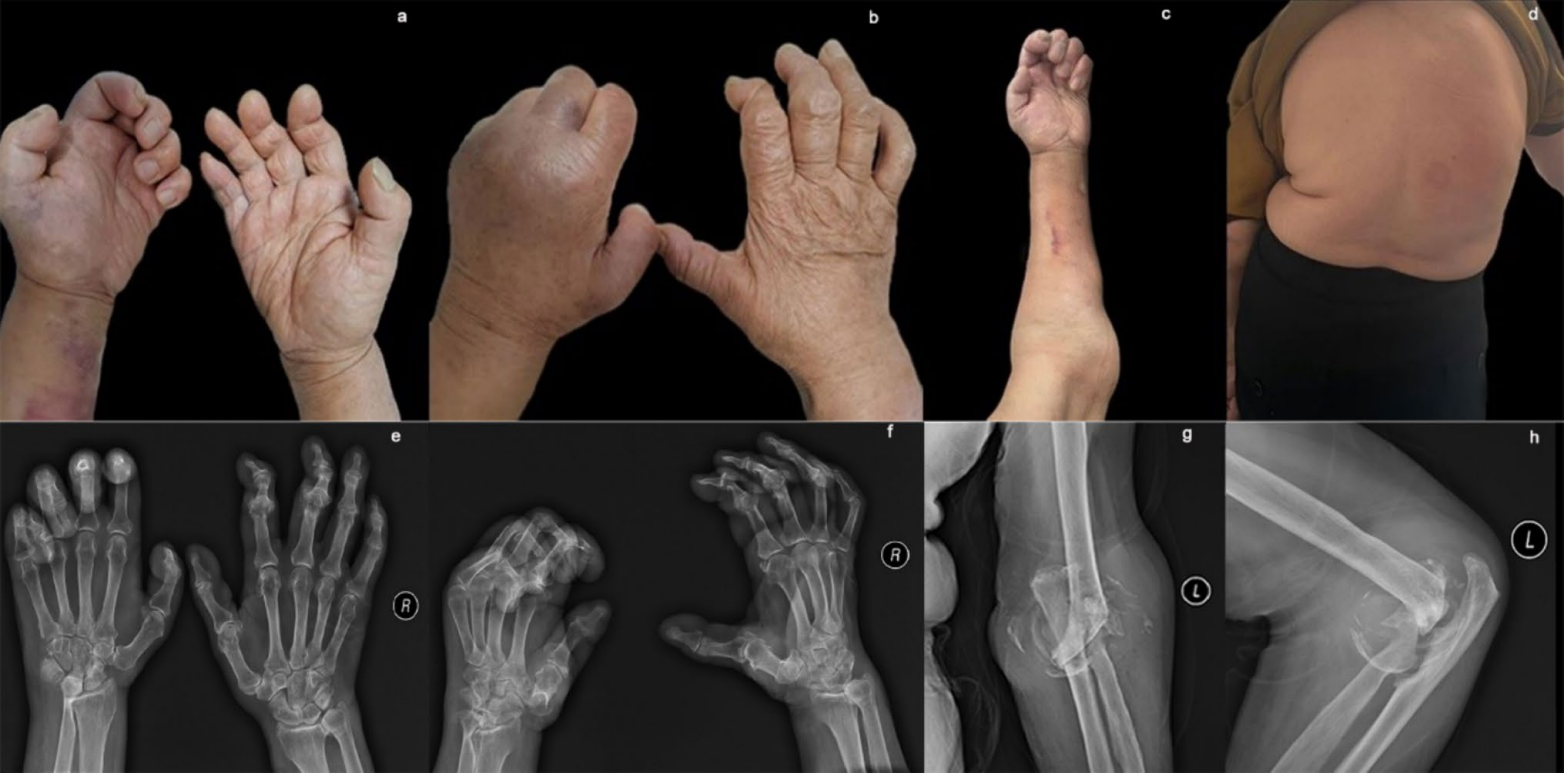
**a**, **b**, **e**, **f** Dysmorphic contracture of the hands; **c**, **g**, **h** a deformed and mutilated elbow; **d** deformity of scoliosis

**Fig. 2:**
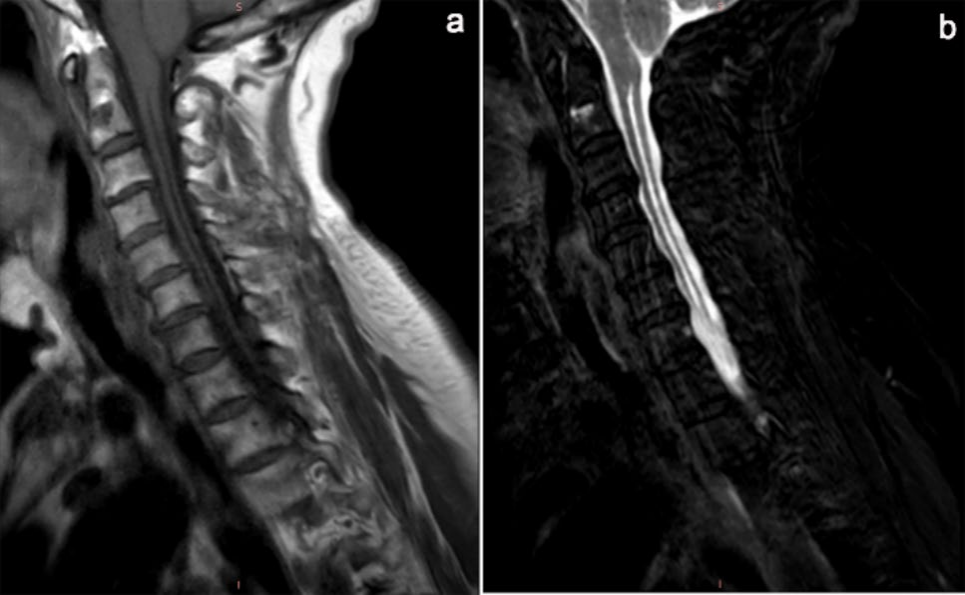
**a**, **b** Cervical spinal cord T1W, T2W: inferior displacement of the cerebellar tonsils. Longitudinal syrinx demonstrating characteristic T1-hypointense and T2-hyperintensesignals

## Data Availability

The datasets analyzed for this study are available from the corresponding author Bing Fan (icii@163.com) upon reasonable request.
